# Deep peroneal neuropathy induced by prolonged squatting: a case report

**DOI:** 10.3389/fnana.2024.1474791

**Published:** 2024-10-09

**Authors:** Hyun-Seok Jo, Ki-Hong Kim, Min-Keun Song, Hyeng-Kyu Park, In Sung Choi, Jae-Young Han

**Affiliations:** ^1^Department of Physical and Rehabilitation Medicine, Chonnam National University Hospital, Gwangju, Republic of Korea; ^2^Department of Physical and Rehabilitation Medicine, Chonnam National University Medical School and Hospital, Gwangju, Republic of Korea

**Keywords:** peroneal nerve, neuropathy, anatomic variation, nerve compression syndromes, ultrasonography

## Abstract

Prolonged squatting is a well-documented cause of common peroneal neuropathy, wherein the common peroneal nerve is thought to be compressed between the biceps femoris tendon and the lateral head of the gastrocnemius muscle or the fibular head. However, deep peroneal neuropathy resulting from prolonged squatting has not been previously reported. We present the case of a tile installer who developed unilateral deep peroneal neuropathy following extended squatting, diagnosed through ultrasonography, which identified the bilateral division of the common peroneal nerves between the knee joint and the fibular head. This case underscores the value of ultrasonography, particularly when electrodiagnostic results are inconsistent with clinical expectations.

## Introduction

Peripheral nerves are susceptible to entrapment within anatomical structures or compression from external pressure, leading to compressive neuropathy. Such compression may be caused by bone, bony callus, synovial thickening, ganglia, tumors, fibrous bands, or muscle (Fabre et al., 1998). Certain areas of the body are anatomically predisposed to compression, with the fibular head being a common site for such occurrences. Common peroneal neuropathy at the fibular head is one of the most frequent compressive neuropathies affecting the lower extremities. The common peroneal nerve wraps around the fibular head and neck, where it adheres closely to the periosteum, rendering it particularly vulnerable to compression (Craig, 2013). This condition can lead to foot drop and tingling pain in the lower extremities, necessitating differential diagnosis from other conditions like radiculopathy (Marciniak, 2013). Therefore, identifying the precise cause of neuropathy is crucial. Established causes include leg-crossing, dilated veins, drastic weight loss, schwannoma, neurofibroma, pneumatic compression, and ganglion cysts (Patel et al., 2013). Prolonged squatting is also recognized as a cause of common peroneal neuropathy, where the nerve is believed to be compressed between the biceps femoris tendon and the lateral head of the gastrocnemius muscle or the fibular head (Babayev et al., 1998). However, deep peroneal neuropathy resulting from prolonged squatting has not been previously reported. We present the case of a tile installer who developed unilateral deep peroneal neuropathy following prolonged squatting.

## Case presentation

**FIGURE 1:**
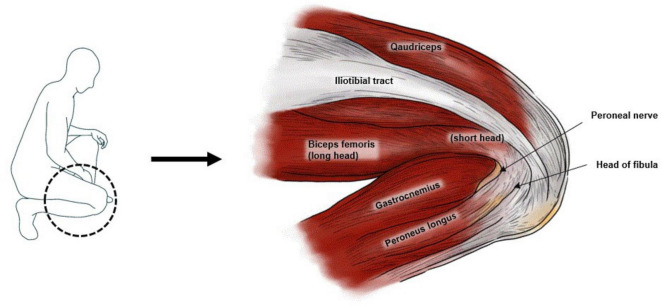
Lateral view of the flexed right knee of the patient, highlighting the anatomical structures surrounding the peroneal nerve.

A 27-year-old man presented with a 3-week history of right foot drop. The onset of symptoms occurred 1 week after he had spent over 8 h squatting and kneeling on his right knee while installing tiles (Figure 1). Seven days into the tile installation, he began experiencing tingling sensations in his right leg. His medical history was unremarkable, with no prior neuropathy or significant family history. He also reported no history of low back pain, and lumbar spine magnetic resonance imaging performed at another hospital was normal. Neurological examination revealed weakness in right ankle dorsiflexion, graded as 1 on the Medical Research Council (MRC) scale, and in right toe dorsiflexion, graded as 3 on the MRC scale. Sensation in the first web space of the right foot dorsum was diminished.

Nerve conduction studies and electromyography were conducted. The nerve conduction study showed normal compound muscle action potentials (CMAPs) in the right peroneal nerve when recorded from the extensor digitorum brevis (EDB) muscle and stimulated at the ankle and popliteal fossa. However, CMAPs recorded from the tibialis anterior (TA) muscle displayed reduced amplitude compared to the contralateral side. Superficial peroneal and sural sensory nerve action potentials were within the normal range. Needle electromyography revealed abnormal spontaneous activities in the right EDB and TA muscles, along with large motor unit action potentials (MUAPs) in the EDB muscle. No abnormalities were found in muscles innervated by the superficial peroneal nerve (Table 1).

**TABLE 1:** Electrodiagnostic study findings.

Nerve	Stimulation	Right			Left		
		Latency (ms)	Amplitude (mV)	CV (m/s)	Latency (ms)	Amplitude (mV)	CV (m/s)
**Motor**							
Peroneal at EDB	Ankle	3.5	14.5	–	3.3	16.5	–
	Below the fibular head	9.9	13.9	48.4	9.0	15.6	54.1
Peroneal at TA	Below the fibular head	3.0	3.1*	–	3.0	7.3	–
	Above the fibular head	4.7	1.0*	58.2	3.9	5.4	64.0
Tibial	Ankle	3.1	35.6		2.7	31.8	
	Popliteal fossa	10.8	30.1	49.9	10.0	27.1	50.7
**Sensory**							
Superficial peroneal	Lateral leg	3.4	17.9		3.4	15.0	
Sural	Calf	3.4	20.9		3.3	19.4	
**Muscle**	**IA**	**ASA**		**MUAP**		**Recruitment pattern**	
Paraspinalis (L3-S1)	Normal	None		Normal		Normal	
Extensor digitorum brevis	Normal	++		Large		Reduced	
Abductor hallucis	Normal	None		Normal		Normal	
Gastrocnemius (medial)	Normal	None		Normal		Normal	
Tibialis anterior	Normal	+		Normal		Reduced	
Peroneus longus	Normal	None		Normal		Normal	
Biceps femoris (short)	Normal	None		Normal		Normal	
Vastus lateralis	Normal	None		Normal		Normal	

CV, conduction velocity; EDB, extensor digitorum brevis; TA, tibialis anterior; IA, insertional activity; ASA, abnormal spontaneous activity; MUAP, motor unit action potential.

* Indicates abnormal values in the nerve conduction study.

**FIGURE 2:**
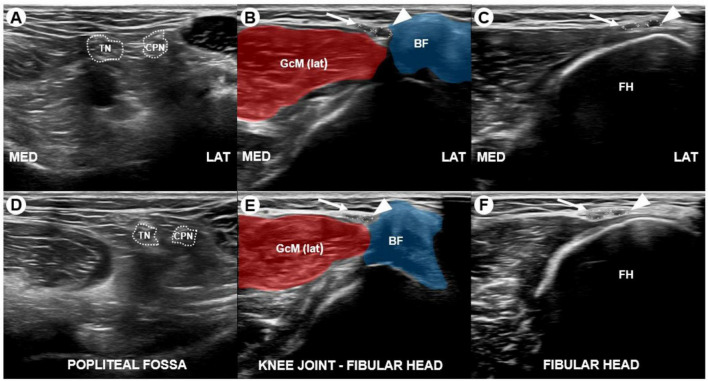
Transverse sonograms of the right **(A–C)** and left **(D–F)** tibial nerve, common peroneal nerve, deep peroneal nerve (arrowhead), and superficial peroneal nerve (white arrow) captured at the popliteal fossa, between the knee joint and fibular head (FH), and at the level of the fibular head (FH). The sonograms reveal bilateral division of the common peroneal nerves between the knee joint and fibular head rather than at the fibular head level, along with hypoechoic swelling of the right deep peroneal nerve. The cross-sectional area (CSA) of the right deep peroneal nerve was 4.2 mm^2^, compared to 2.2 mm^2^ for the left deep peroneal nerve. TN, tibial nerve; CPN, common peroneal nerve; BF, biceps femoris; GcM (lat), lateral head of gastrocnemius; FH, fibular head.

Given that our findings differed from the common peroneal neuropathy associated with squatting reported in the literature, we performed additional ultrasonography. Ultrasonographic evaluation revealed that, bilaterally, the common peroneal nerves divided between the knee joint and the fibular head rather than at the fibular neck. Marked swelling of the right deep peroneal nerve was observed at the fibular head level, while the left deep peroneal nerve showed no abnormal echogenicity or swelling (Figure 2). Consequently, we diagnosed the patient with right deep peroneal neuropathy and initiated rehabilitation, including range of motion exercises, strengthening exercises, and electrical stimulation therapy.

Three months later, the patient returned for a follow-up visit, during which a repeat electrodiagnostic study was performed. The CMAPs recorded from the TA muscle had improved, showing no side-to-side difference. However, abnormal spontaneous activities persisted in the right EDB and TA muscles on needle electromyography. High amplitude MUAPs and polyphasic MUAPs indicate reinnervation through collateral sprouting (Preston and Shapiro, 2002). Additionally, large MUAPs were noted in the right EDB, and polyphasic MUAPs were observed in the right TA muscle, suggesting ongoing nerve regeneration. Motor strength in the right ankle and great toe had improved to MRC grade 4.

## Discussion

Our report highlights a case of unilateral deep peroneal neuropathy resulting from prolonged squatting in a tile installer. The common peroneal nerve is particularly vulnerable to injury due to its superficial course at the level of the fibular head (Bonfiglioli et al., 2015). Consequently, positions such as sitting cross-legged and lying down are known to precipitate common peroneal neuropathy (Yu et al., 2013). Squatting is also recognized as a risk factor, and several studies have documented cases of common peroneal neuropathy associated with this posture (Kodaira et al., 2017; Toğrol, 2000; Kumar et al., 2021). Based on the patient’s history and physical examination, we initially diagnosed common peroneal neuropathy due to prolonged squatting. Previous studies have reported bilateral common peroneal neuropathy following squatting (Kodaira et al., 2017; Toğrol, 2000; Yildirim and Temel, 2023). However, given that our patient presented with unilateral symptoms, we asked him to replicate the posture he adopted while installing tiles. It became clear that only his right knee had been kneeling, explaining the unilateral presentation. Additionally, the absence of recurrent symptoms and a notable family history ruled out further evaluation for hereditary neuropathy with liability to pressure palsies.

The nerve conduction study and needle electromyography suggested injury confined to the deep peroneal nerve, while the superficial peroneal nerve remained intact. Typically, isolated injury of the deep peroneal nerve can be suspected as anterior tarsal tunnel syndrome, where entrapment occurs under the extensor retinaculum at the ankle (Andresen et al., 1992). However, this possibility was excluded in this patient based on electrodiagnostic studies, which revealed abnormal findings in the TA muscle. Electrodiagnostic studies are instrumental in localizing peripheral nerve lesions and predicting prognosis (Huynh and Kiernan, 2011). But in our nerve conduction study, the EDB muscle was only mildly affected, but the TA muscle was severely affected. Previous studies suggested that the nerve fibers to TA muscle are located most medially in the internal fascicular anatomy of peroneal nerve (Sunderland, 1978). This anatomical positioning may explain why the TA muscle was severely affected. Also, the difference in conduction velocity on the affected side was not prominent. Generally, persistent compression may eventually lead to axonal loss (Malik et al., 2023). Also, in compression neuropathies, various grades of peripheral nerve injury, such as neuropraxia, which can causes conduction blocks can occur (Drăghici et al., 2023). In axonal loss lesions, conduction velocity and distal latency can be normal, provided that the largest and fastest conducting axons remain intact (Preston and Shapiro, 2020). In our case, conduction velocity on the affected side was slightly decreased, suggesting that fast conducting axons were somewhat preserved. However, other axons may have experienced demyelination. Therefore, checking CMAP duration could be a useful method. Therefore, determining the lesion site was challenging. However, in our case, the conduction velocity differences on the affected side were not significant, making it challenging to precisely localize the lesion. Foot drop can also result from other conditions such as sciatic mononeuropathy, lumbosacral plexopathy, or radiculopathy (Van Langenhove et al., 1989). Therefore, we employed ultrasonography as an additional diagnostic modality. A prior study found that approximately 81% of individuals exhibit bifurcation of the common peroneal nerve at or below the level of the fibular neck, with 9% bifurcating between the fibular neck and the knee joint and 10% above the knee joint line (Van den Bergh et al., 2013). In our patient, we observed that the common peroneal nerve bifurcated between the knee joint and the fibular head, underscoring the value of ultrasonography as a diagnostic tool. Ultrasonography offers advantages such as being cost-effective and relatively non-invasive. Actually, we performed needle electromyography on multiple muscles in the patient, which can cause discomfort and may affect the compliance with the examination. Moreover, even if nerve conduction study results are normal, ultrasonography can provide critical diagnostic information by measuring the cross-sectional area of the nerve when symptoms are present (Chen and Fowler, 2022). In this case, we identified swelling in the affected deep peroneal nerve by comparing the cross-sectional area to the unaffected side. We also compared the diagnostic cut-off values with those reported in previous studies (Kim et al., 2016).

Anatomic variations can lead to misinterpretation during electrodiagnostic studies, potentially altering the course of treatment (Tankisi et al., 2014). Thus, accurate diagnosis is crucial, and ultrasonography, which can detect structural abnormalities or anatomical variations in real time, is a valuable complementary tool. In our case, the combination of electrodiagnostic studies and ultrasonography was particularly beneficial, especially given the anatomical variation observed.

After an accurate diagnosis using ultrasonography and electrodiagnostic studies, we prescribed range of motion exercises, strengthening exercises and electrical stimulation therapy for foot drop. Additionally, considering an ankle-foot orthosis could enhance stability during gait and help prevent falls. Also, medications such as pregabalin can be prescribed for the patient’s neuropathic pain, such as tingling sensation (Derry et al., 2019).

In conclusion, when electrodiagnostic results are inconsistent with clinical expectations, ultrasonographic evaluation is necessary to identify structural abnormalities or anatomical variations in the surrounding nerves. This approach facilitates accurate diagnosis and enables more targeted treatment for the patient.

## Data Availability

The original contributions presented in this study are included in this article/supplementary material, further inquiries can be directed to the corresponding author.

## References

[B1] Andresen B. Wertsch J. Stewart W. (1992). Anterior tarsal tunnel syndrome. *Arch. Phys. Med. Rehabil.* 73 1112–1117. 1444780

[B2] Babayev M. Bodack M. Creatura C. (1998). Common peroneal neuropathy secondary to squatting during childbirth. *Obstet. Gynecol.* 91 830–832. 9572179 10.1016/s0029-7844(97)00717-5

[B3] Bonfiglioli R. Mattioli S. Violante F. (2015). Occupational mononeuropathies in industry. *Handb. Clin. Neurol.* 131 411–426. 26563800 10.1016/B978-0-444-62627-1.00021-4

[B4] Chen J. Fowler J. (2022). Ultrasound findings in patients with normal nerve conduction despite clinical signs and symptoms consistent with carpal tunnel syndrome. *Plast Reconstr. Surg.* 150 1025e–1032e. 10.1097/PRS.0000000000009622 35998137

[B5] Craig A. (2013). Entrapment neuropathies of the lower extremity. *PM R.* 5 S31–S40. 23542774 10.1016/j.pmrj.2013.03.029

[B6] Derry S. Bell R. Straube S. Wiffen P. Aldington D. Moore R. (2019). Pregabalin for neuropathic pain in adults. *Cochrane Database Syst. Rev.* 2019:CD007076. 10.1002/14651858.CD007076.pub3 PMC6353204 30673120

[B7] Drăghici N. C. Văcăraş V. Bolchis R. Bashimov A. Domniţa D. M. Iluţ S. (2023). Diagnostic approach to lower limb entrapment neuropathies: A narrative literature review. *Diagnostics* 13:3385. 10.3390/diagnostics13213385 PMC10647627 37958280

[B8] Fabre T. Piton C. Andre D. Lasseur E. Durandeau A. (1998). Peroneal nerve entrapment. *J. Bone Joint Surg. Am.* 80 47–53. 9469308 10.2106/00004623-199801000-00009

[B9] Huynh W. Kiernan M. (2011). Nerve conduction studies. *Aust. Fam. Phys.* 40 693–697. 21894276

[B10] Kim J. Song S. Park H. Rhee W. Won S. (2016). Diagnostic cutoff value for ultrasonography of the common fibular neuropathy at the fibular head. *Ann. Rehabil. Med.* 40 1057–1063. 28119836 10.5535/arm.2016.40.6.1057 PMC5256328

[B11] Kodaira M. Sekijima Y. Ohashi N. Takahashi Y. Ueno K. Miyazaki D. (2017). Squatting-induced bilateral peroneal nerve palsy in a sewer pipe worker. *Occup. Med.* 67 75–77. 10.1093/occmed/kqw133 27694375

[B12] Kumar H. Shah S. Kor J. Qazi S. Kumar J. (2021). Common peroneal neuropathy in harvesting farmers. *Pak. J. Neurol. Sci.* 16 6–9.

[B13] Malik T. Malik A. Abd-Elsayed A. (2023). Pathophysiology of work-related neuropathies. *Biomedicines* 11:1745. 10.3390/biomedicines11061745 PMC10296724 37371842

[B14] Marciniak C. (2013). Fibular (peroneal) neuropathy: Electrodiagnostic features and clinical correlates. *Phys. Med. Rehabil. Clin. N. Am.* 24 121–137. 23177035 10.1016/j.pmr.2012.08.016

[B15] Patel A. Singh R. Johnson B. Smith A. (2013). Compression neuropathy of the common peroneal nerve by the fabella. *BMJ Case Rep.* 2013:bcr2013202154. 10.1136/bcr-2013-202154 PMC3847518 24293541

[B16] Preston D. Shapiro B. (2002). Needle electromyography. Fundamentals, normal and abnormal patterns. *Neurol. Clin.* 20 361–96, vi. 10.1016/s0733-8619(01)00005-6 12152440

[B17] Preston D. Shapiro B. (2020). *Electromyography and neuromuscular disorders e-book: Clinical-electrophysiologic-ultrasound correlations.* Amsterdam: Elsevier Health Sciences, 811.

[B18] Sunderland S. (1978). *Nerves and nerve injuries.* London: Churchill Livingstone, 1068.

[B19] Tankisi H. Pugdahl K. Otto M. Fuglsang-Frederiksen A. (2014). Misinterpretation of sural nerve conduction studies due to anatomical variation. *Clin. Neurophysiol.* 125 2115–2121. 10.1016/j.clinph.2014.01.030 24618219

[B20] Toğrol E. (2000). Bilateral peroneal nerve palsy induced by prolonged squatting. *Mil. Med.* 165 240–242. 10741091

[B21] Van den Bergh F. Vanhoenacker F. De Smet E. Huysse W. Verstraete K. (2013). Peroneal nerve: Normal anatomy and pathologic findings on routine MRI of the knee. *Insights Imaging* 4 287–299. 10.1007/s13244-013-0255-7 23709403 PMC3675257

[B22] Van Langenhove M. Pollefliet A. Vanderstraeten G. (1989). A retrospective electrodiagnostic evaluation of footdrop in 303 patients. *Electromyogr. Clin. Neurophysiol.* 29 145–152. 2721427

[B23] Yildirim A. Temel M. (2023). Bilateral peroneal nerve palsy due to prolonged squatting in farmers: Clinical and electrophysiological outcome. *Neurol. Res.* 45 118–123. 10.1080/01616412.2022.2124793 36111735

[B24] Yu J. Yang J. Kang S. Cho Y. (2013). Clinical characteristics of peroneal nerve palsy by posture. *J. Korean Neurosurg. Soc.* 53 269–273. 10.3340/jkns.2013.53.5.269 23908699 PMC3730027

